# Gametic cycle and gonadal maturity of *Bulimulus bonariensis* (Rafinesque, 1833) (Gastropoda: Bulimulidae), in a rural area of Buenos Aires, Argentina

**DOI:** 10.7717/peerj.15221

**Published:** 2023-04-18

**Authors:** Ana Carolina Díaz, Stella Maris Martin, Alejandra Rumi

**Affiliations:** 1 División Zoología Invertebrados, Facultad de Ciencias Naturales y Museo, Universidad Nacional de La Plata, La Plata, Buenos Aires, Argentina; 2 CIC, La Plata, Buenos Aires, Argentina; 3 CONICET, La Plata, Buenos Aires, Argentina

**Keywords:** Gonadal stages, Size at gonadal maturity, Reproductive biology

## Abstract

*Bulimulus bonariensis*, a snail distributed over a large part of Argentina, has generated negative effects on the agribusiness. We accordingly conducted a seasonal sampling during 2018–2019 in order to characterize the gametic cycle and establish the size of this gastropod at gonadal maturity. Three reproductive stages were identified: the mature in autumn; the spawn, which begins in winter but occurs mainly in the spring, followed by the post-spawn in summer, where an absence of gonadal rest was evidenced. In the fall, in maturity resorption was observed in addition to abundant primary spermatocytes, secondary spermatocytes, and growing vitellogenic oocytes. At the same time, could correlate the degree of spermioviduct development with the stage of gonadal development. Moreover, we used a logistic regression to calculate the size at gonadal maturity, which was established at the total shell length of 12 mm. In addition, we found that at the beginning of reproductive development those gastropods are protogynous hermaphrodites, but after reaching gonadal maturity became simultaneous hermaphrodites. Finally, *B. bonariensis* also proved to be an iteroparous species. The information provided here will be essential for delineating and establishing population control strategies.

## Introduction

The family Bulimulidae Tryon, 1867 comprises about 543 valid species (MolluscaBase, 2022) native to the tropics and subtropics of South America, Australia, New Zealand, Tasmania, and Africa (Breure, 1979; Herbert & Mitchell, 2009). Especially in the Argentine Republic, this family is the richest and most phylogenetically diverse, as it includes five genera with 46 species (Cuezzo, Miranda & Ovando, 2013; Miranda, 2014; Miranda & Cuezzo, 2014). Within Argentina, eight species in *Bulimulus* Leach, 1814 have been described, among which *Bulimulus bonariensis* is the most widely distributed, extending over twelve provinces: Buenos Aires, Chaco, Córdoba, Corrientes, Entre Ríos, Formosa, Jujuy, Misiones, Salta, Santiago del Estero, Santa Fe, and Tucumán (Miquel, 1991). *B. bonariensis* is a species of significance in the agribusiness because of it is abundance in soybean fields, where it has caused a clogging of harvesting machinery and a loss of grain (Frana & Massoni, 2007; Frana & Massoni, 2011). In yerba-mate crops, the leaves have been rendered unusable because of the mucus left behind by the snail (Noticias La Región, 2013). In addition, it has exerted negative effects on chickpea grains, Peralta (2016) cataloged this species as a pest causing indirect damage.

Therefore, information related to gonad activity is needed to complement the present understanding of the species’s life cycle, reproductive biology, dynamics, and population structure. Information that could be applied to the strategies of population control by predicting the breeding periods. In Argentina only one study of this type that has been reported for the family (Cuezzo, 1993) for the specie *Scutalus tupacii* (d’Orbigny, 1835). With respect to terrestrial gastropods, the investigation related to gametic cycles is scarce and refers to species phylogenetically distant from *B. bonariensis*, as *Arion ater* (Linnaeus, 1758) (Arionidae), *Lissachatina fulica* (Bowdich, 1822) (Achatinidae), *Neohelix major* (Binney, 1837) (Polygyridae), *Helix pomatia* Linnaeus, 1758 (Helicidae), *Helicodonta obvoluta* (O.F. Müller, 1774) (Helicodontidae), *Megalobulimus abbreviatus* (Bequaert, 1948) (Strophocheilidae), *Vertigo pusilla* (O.F. Müller, 1774) (Vertiginidae), *Vestia* Hesse, 1916 (Clausiliidae), *Oxychilus (Atlantoxychilus) spectabilis* (Milne-Edwards, 1885) (Oxychilidae), *Cerion mumia chrysalis* (Férrusac, 1837) (Cerionidae) (Parivar, 1978; Ngowsiri et al., 1989; Cuezzo, 1990; Juchno, 1999; Maltz, 2003; Horn, Achaval & Zancan, 2005; Mazurkiewicz & Pokryszko, 2005; Maltz & Sulikowska-Drozd, 2010; Ferreira et al., 2012; Suarez, 2015).

This work aims to contribute with information on the reproductive strategy of *B. bonariensis,* from an analysis of the gametic cycle and the identification of the size of the gastropod at gonadal maturation, through histological criteria from individuals obtained over the course of two years.

## Materials & Methods

### Sampling

**Figure 1:**
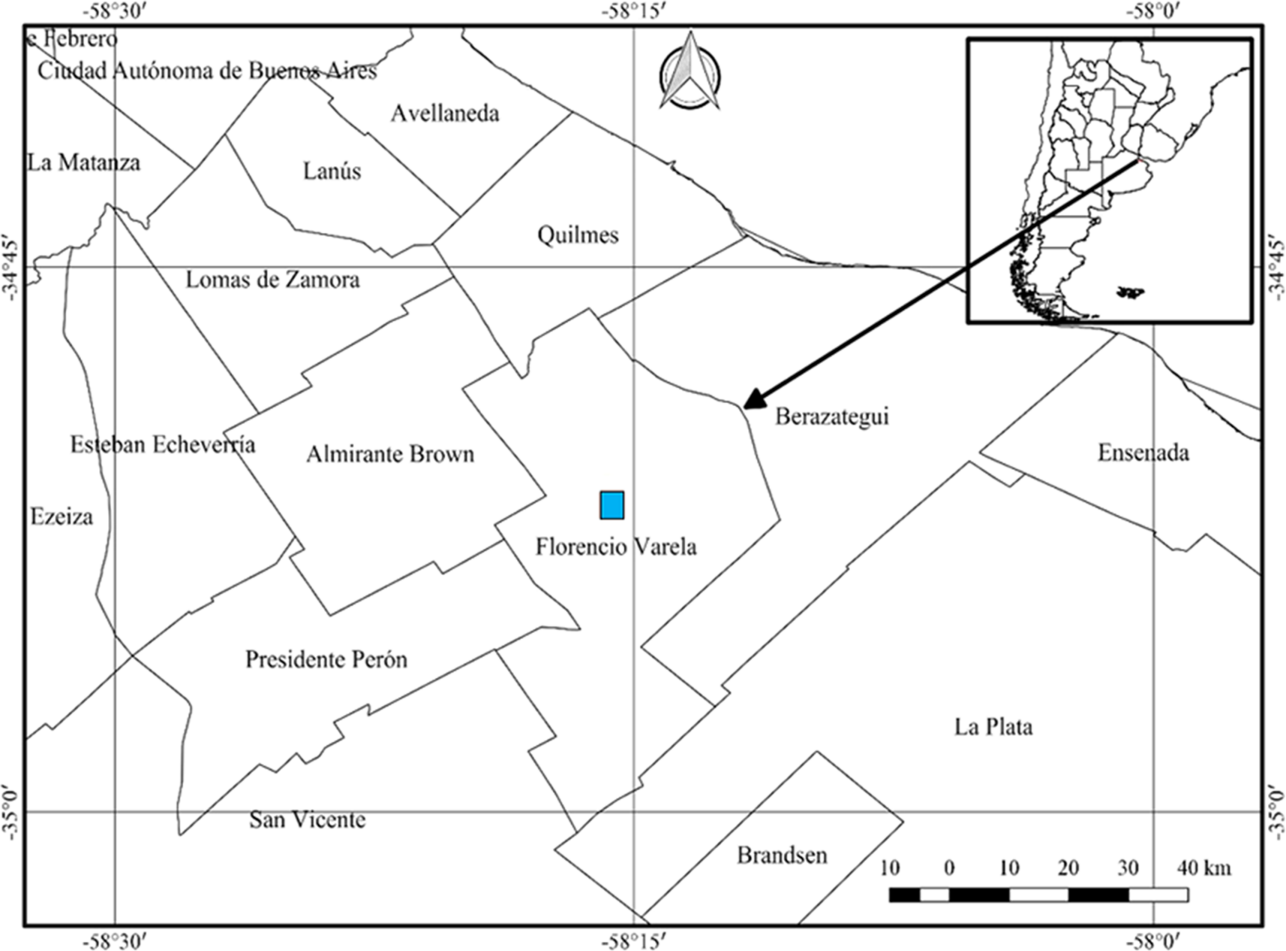
Location of the sampling site for obtaining *Bulimulus bonariensis* specimens for the study of the gametic cycle and gonadal maturity. Florencio Varela lies adjacent to the West side of La Plata, with the edge of the autonomous city of Buenos Aires lying in the upper left corner of the figure. The inset and arrow indicate the geography of the sampling site within Argentina. Image credit: Díaz Ana Carolina.

**Figure 2:**
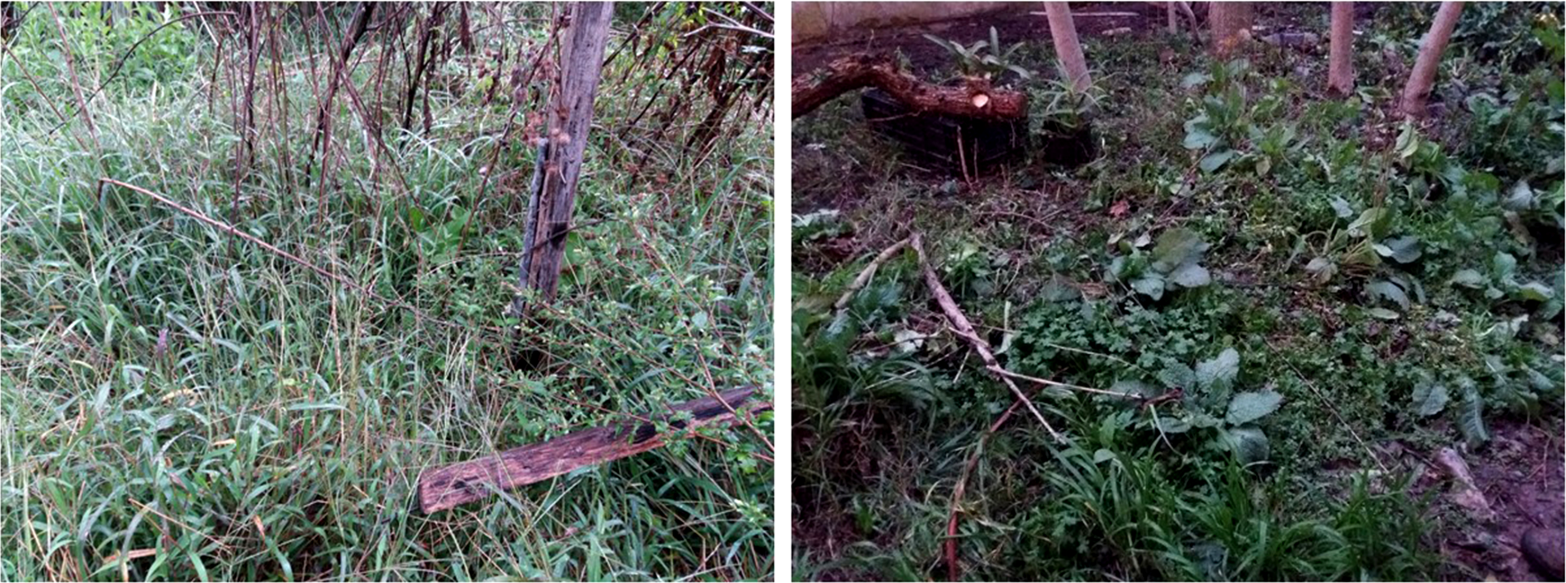
The environment from which the *Bulimulus bonariensis* samples were taken. Photo credit: Díaz Ana Carolina.

The study was conducted over the course of two consecutive years (March 2018 through December 2019), in a private field in the rural area of Villa San Luis belonging to the town of Florencio Varela (34°51′7.617″S, 58°15′53.932″W), province of Buenos Aires (Fig. 1). Authorization granted by note under docket number Cod 100 No 6359. The sampling was carried out in the early hours of the morning or at night in periods after rainfall or during drizzles, since the high humidity of the environment enables gastropods to be more active (Barrientos, 2003). The sampling area was delimited following De Winter & Gittenberger (1998) with modifications. Three 15 ×15 meter plots were demarcated in the field which were rotated in the different collection dates. The material was collected manually for 2 h each time, after a thorough checking of all the microhabitats in the plot. Figure 2 illustrates the sampling environment. In addition, climatological information was obtained from the National Meteorological Service (Servicio Meteorológico Nacional) available at https://www.smn.gob.ar/.

### Processing

The samples obtained were transported to the laboratory following the recommendations of Barrientos (2003). The specimens were relaxed and sacrificed by placing them in airtight containers with water and Parafarm^®^ menthol crystals for 12–16 h. The separation of soft tissue was performed under a Leica EZ4 stereo microscope. The total length of the shell was measured with a millimetric vernier caliper (precision of 0.01 mm). After the dissection and anatomical observation, the penile complex was measured under the stereo microscope with a micrometer eyepiece from the base of the penile sheath to the end of the flagellum.

For histological analysis, the gonad was fixed in Raillet-Henry’s solution for 12 h and preserved in 70% (v/v) aqueous ethanol followed by dehydration in an increasing sequence of alcohols. For inclusion, the tissue was immersed in Histoplast^®^, slices of 7 µm were made with a Minot-type microtome and stained with Mayer’s hematoxylin-eosin before mounting in synthetic balsam. The histological preparations were observed and photographed under a Leica-DMLS light microscope.

### Gametogenic cycle

The establishment of the different gonadal stages as the basis of descriptive qualitative data was performed upon observation of the histological preparations. The gonadal cycle was divided into five stages: proliferation, growth, pre-evacuation, evacuation, and resorption; and in order to complement the description of the gametic cycle, a classification of the germ-cell population was made according to the criteria proposed by Maltz & Sulikowska-Drozd (2010). Accordingly, the cell types were considered as follows: 0, absence of a cell type; 0.5, with 1-2 cell clusters or 1-2 oocytes; 1, several cell clusters or 3-6 oocytes; 2, more than 10 cell clusters or more than 6 oocytes. In addition, to determine the stages of female germ-line development, the oocyte diameters, as estimated after Griffond & Bolzoni-Sungur (1986), were recorded after measurement by the digital-analysis program FIJI. (available at https://fiji.sc/). The reproductive stages were established in accordance with the terminology used in Giese & Pearse (1977) and Baqueiro Cárdenas (2014).

### Size at first maturity

To determine the minimum size of the snails at initial gametogenesis, individuals were classified according to the state of the ovotestis: 0, individuals with undifferentiated or immature gonads involving the presence of undifferentiated germ cells and previtellogenic oocytes, and 1, mature individuals exhibiting the presence of vitellogenic oocytes and spermiogenesis stages. The data were then pooled every 0.5 mm and the proportion of gonadal maturity calculated. A logistic regression, through the use of the PAST 3.25 program (available at https://past.en.lo4d.com/windows), was used to calculate the body length at which 50% of the individuals were mature. Finally, the fit of the model was evaluated with SPSS Statistics version 22 (available at https://www.ibm.com/products/spss-statistics).

In addition, the relationship between total penile complex length and total body length was evaluated by a simple regression analysis to determine if the latter measurement could be considered another maturity parameter. Furthermore, the general appearance of the reproductive system was observed in order to determine if a morphologic variation occurred in any structure throughout an annual gonadal cycle.

## Results

### Sampling

The study site is characterized by a temperate humid subtropical climate. Table 1 shows the environmental variables, temperature and precipitation, by season for the years studied.

Material was obtained from 15 samplings involving 168 individuals in total, which were grouped by season. In 2018, 88 individuals were collected 21 (summer), 25 (autumn), 14 (winter), 28 (spring), and in 2019, 80 individuals 24 (summer), 26 (autumn), 13 (winter), 17 (spring).

### Cell types

**Figure 3:**
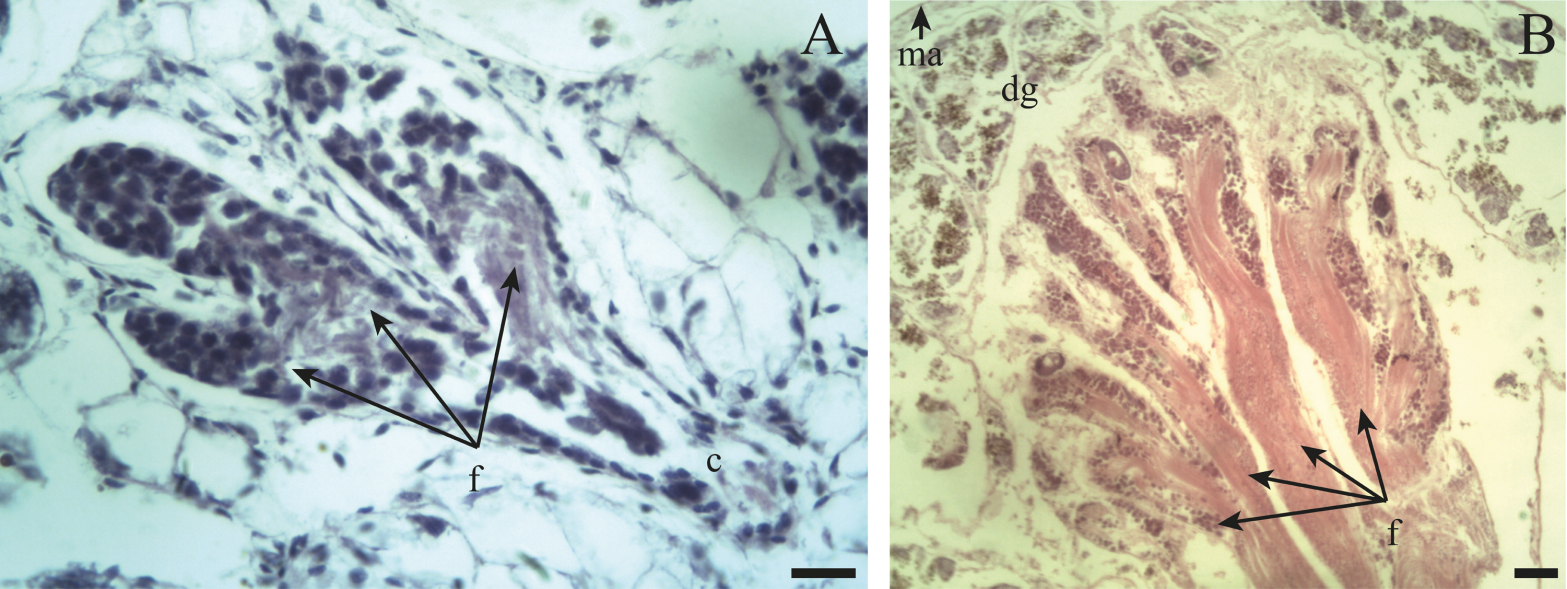
General appearance of the ovotestis of *Bulimulus bonariensis*. (A) Follicles (f) and duct (c) of the ovotestis in the undifferentiated stage of development. Scale bar = 20 µm. (B) Appearance of the ovotestis in the differentiated stage of development, evidencing the parallel arrangement of the follicles (f), the digestive gland (dg) surrounding the gonad and the mantle (ma). Scale bar = 50 µm. Photo credit: Díaz Ana Carolina.

The ovotestis in an immature (Fig. 3A) or mature (Fig. 3B) state, is formed by parallel acini or follicles grouped in lobes one after the other.

**Figure 4:**
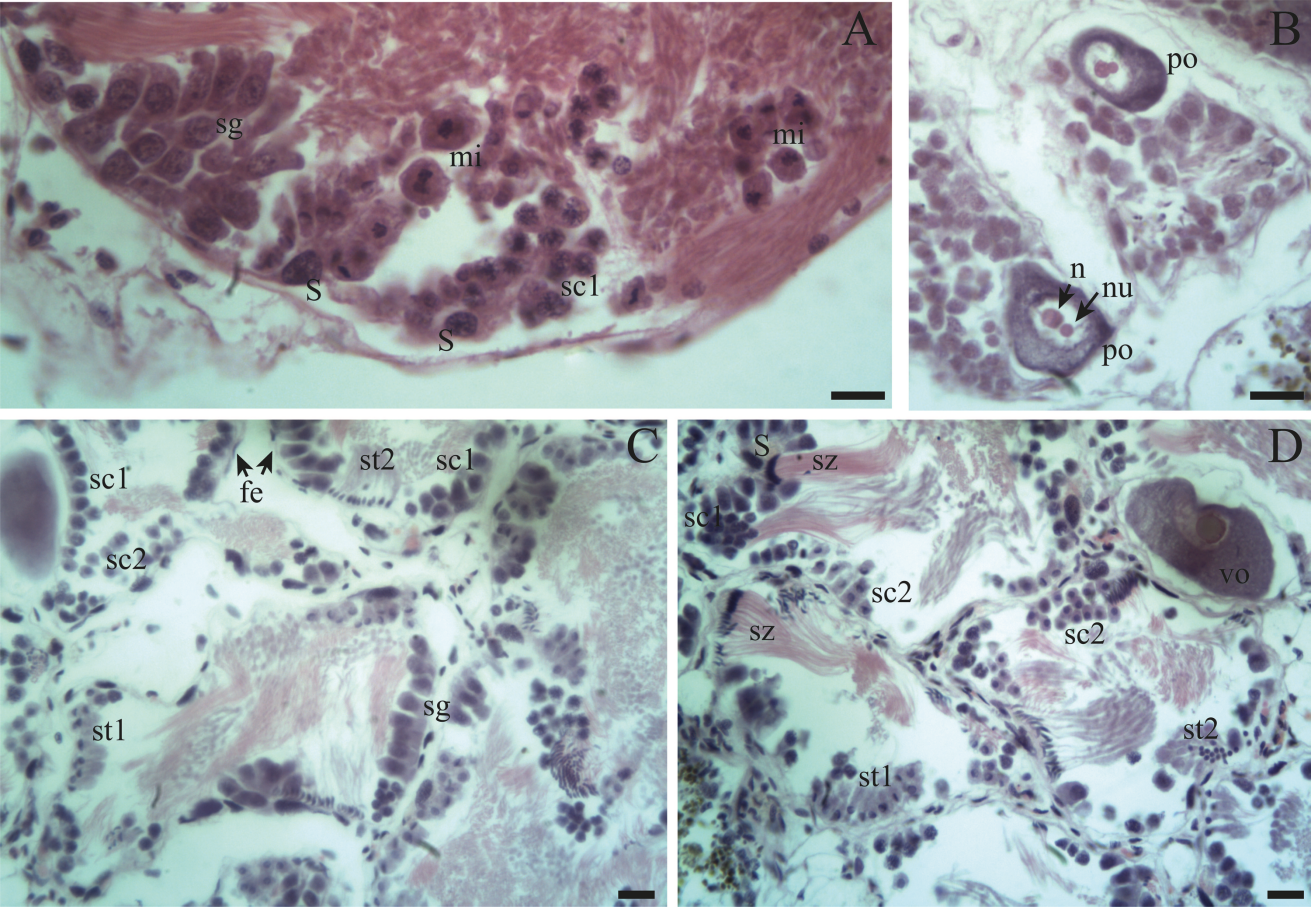
Gonadal stages of *Bulimulus bonariensis* during proliferation and growth. (A) Appearance of a proliferating follicle with abundant mitoses (mi), and the presence of spermatogonia (sg), primary spermatocytes (sc1) and Sertoli cells (S). (B) Morphology of previtellogenic oocytes (po) in growth. The nucleus (n) and the nucleolus (nu) are differentiated. (C–D) Initial growth and in full development respectively: spermatogonia (sg) are observed along with primary spermatocytes (sc1), secondary spermatocytes (sc2), early spermatids (st1), late spermatids (st2), and mature spermatozoa (sz) associated with the follicular epithelium (fe). The female germ line was characterized by the presence of vitellogenic oocytes (vo). Scale bar = 20 µm. Photo credit: Díaz Ana Carolina.

**Figure 5:**
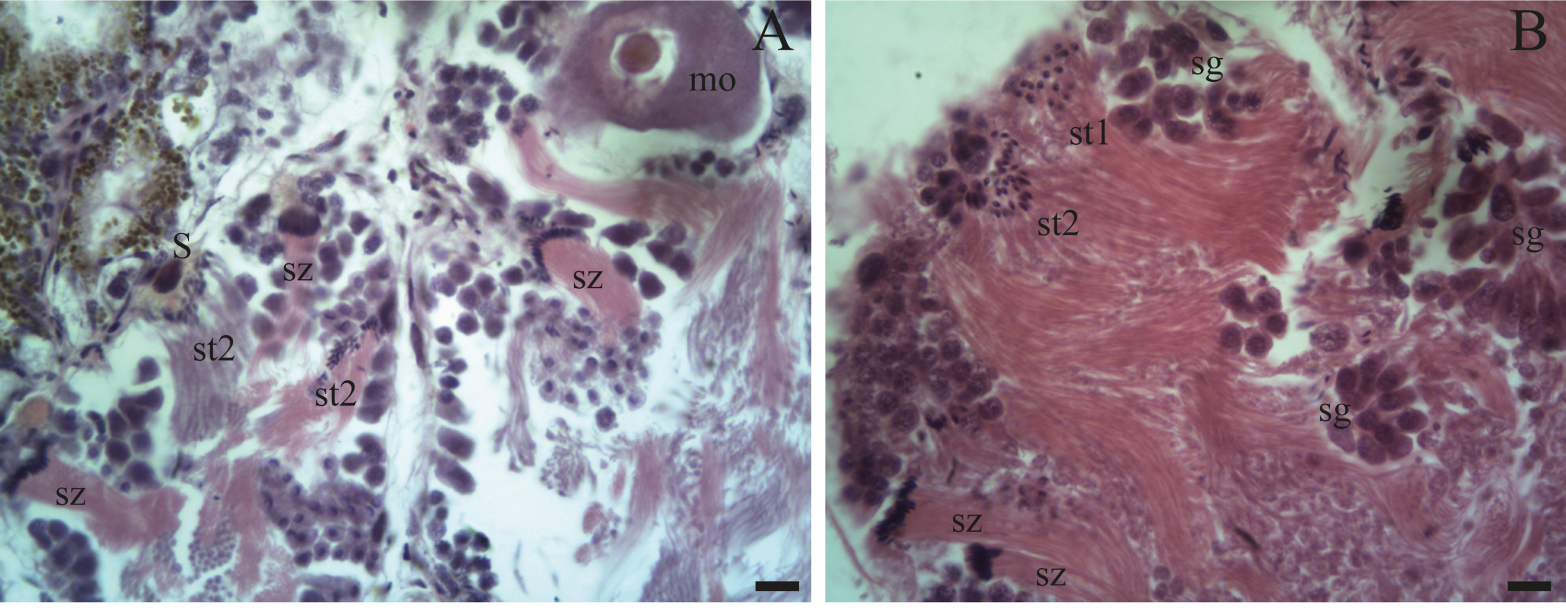
Gonadal stage of *Bulimulus bonariensis* during pre-evacuation. (A–B) The female germ line only contains mature oocytes (mo); while the male germ line manifests spermatogonia (sg), early spermatids (st1), late spermatids (st2), and abundant spermatozoa clusters (sz) surrounding the Sertoli cells (S). Scale bar = 20 µm. Photo credit: Díaz Ana Carolina.

**Figure 6:**
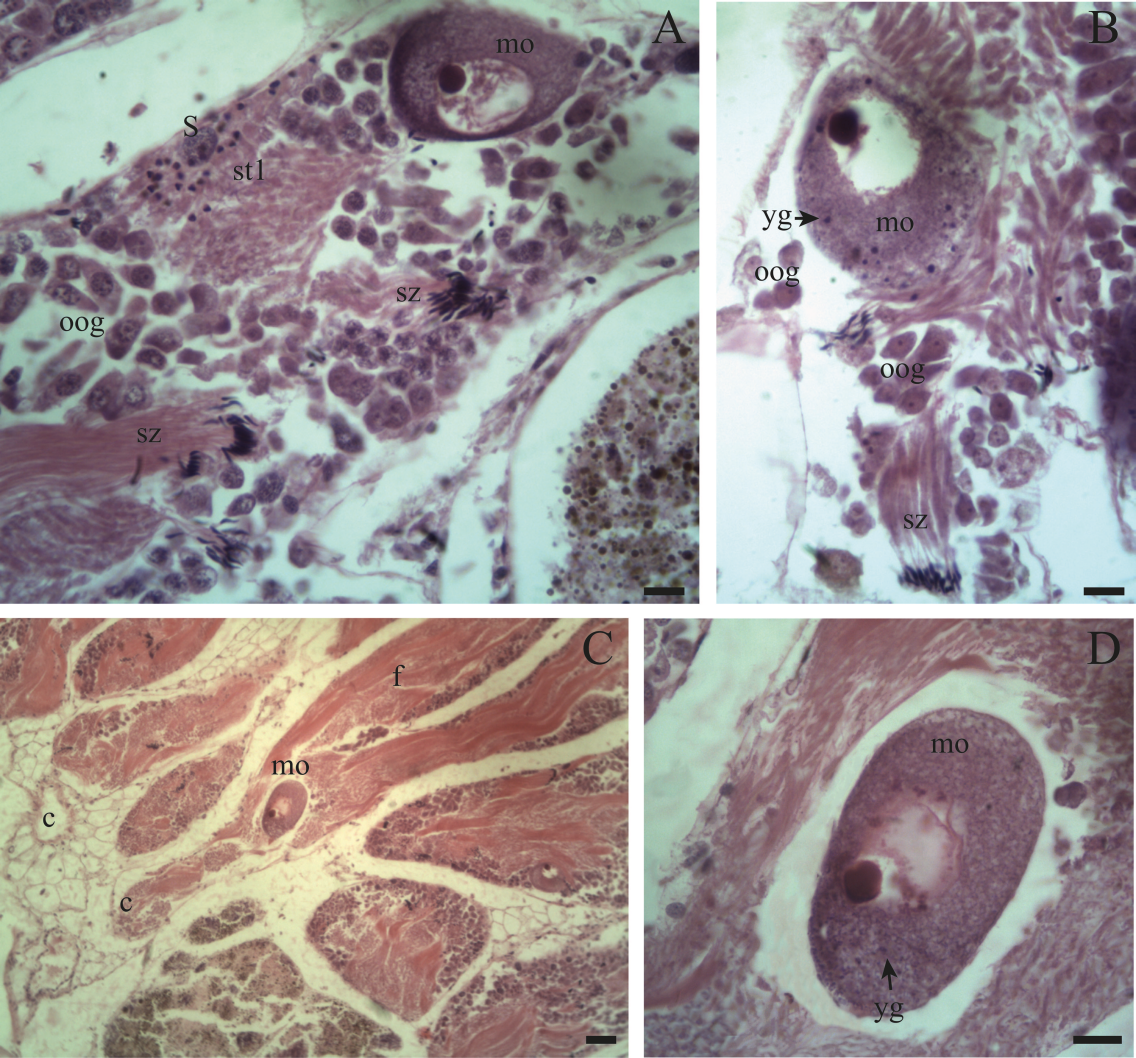
Gonadal stage of *Bulimulus bonariensis* during evacuation. (A–B) With some early spermatids (st1), spermatozoa clusters (sz), and mature oocytes (mo); where the first oogonia (oog) appear. (C–D) Mature oocyte with yolk granules (yg) moving from the follicle (f) of origin towards the duct (c). Scale A = 50 µm, B–D = 20 µm. Photo credit: Díaz Ana Carolina.

**Figure 7:**
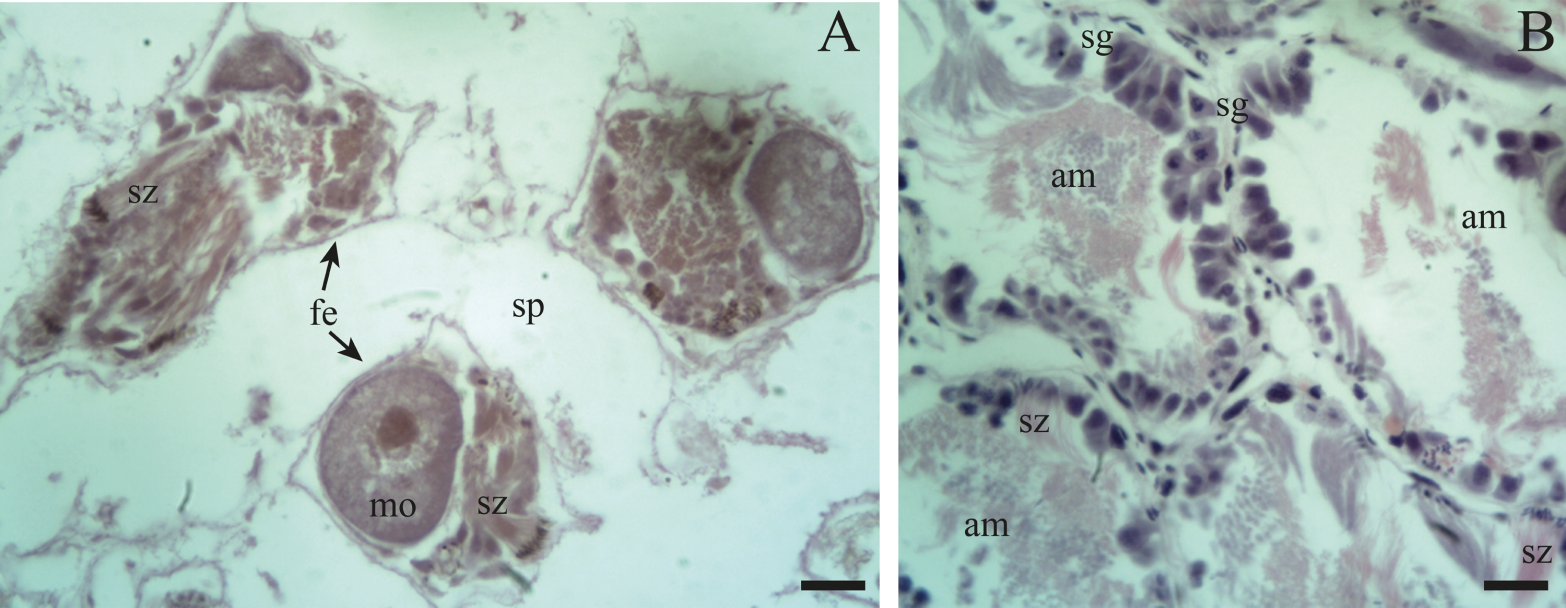
Gonadal stage of *Bulimulus bonariensis* during resorption. (A) The follicles are with wide spaces (sp) between follicular epithelia (fe), though still containing some sperm clusters (sz) and mature oocytes (mo). (B) Reproductive debris is present in the lumen of the follicles and is degraded by amaebocytes (am) with some spermatozoa clusters (sz) remaining. The proliferation of spermatogonia (sg) begins. Scale bar = 20 µm. Photo credit: Díaz Ana Carolina.

Spermatogenesis develops associated to the epithelium that limits the follicle. As spermiogenesis proside, the developing sperms become oriented towards the lumen. Quadrangular shaped spermatogonia ∼20 µm long were identified with large ∼7 to 8 µm nuclei and abundant cytoplasm, arranged in clusters (Figs. 4A; 5B: sg) or parallel to each other (Fig. 4C) in direct contact with the follicle epithelium. The primary spermatocytes are rounded (7–10 µm in diameter), and the chromatin is dispersed occupying the cell body almost completely (Figs. 4A, 4C, 4D: sc1). The secondary spermatocytes have a rounded shape and a diameter similar to the primary spermatocytes, but the chromatin is condensed in the center of the cell, and the cytoplasm is differentiated (Figs. 4C, 4D: sc2). The spermatids are present two stages: the early spermatids have an elongated cell body with a round nucleus located at the end of the cell (Figs. 4C, 4D; 5B; 6A: st1). The cells are in contact with the epithelium of the follicle or are located surrounding the Sertoli cells, which provide support and nutrition (Figs. 4A, 4D; 5A; 6A: S). In the late spermatids the nucleus becomes oval in shape and the tail begins to differentiate (Figs. 4C, 4D, 5A, 5B: st2). The mature spermatozoa have a defined head, they form clusters with their long tails or flagella directed towards the follicle lumen, and are located in contact with the follicular wall or a Sertoli cell or lie in the follicle lumen ready to descend through the ducts (Figs. 4D; 5A, 5B, 6A, 6B; 7A, 7B: sz).

**Table 1:** Seasonal variation of temperature and seasonal precipitation in Florencio Varela, Buenos Aires (2018–2019).

Season	Year	Mean temperature (°C)	Mean maximum temperature (°C)	Mean minimum temperature (°C)	Mean precipitation (mm)
Summer	‘18	25	37	12	50
	‘19	24	37	11	112.5
Autumn	‘18	19	31.6	7.6	200
	‘19	18.2	31	8.3	75
Winter	‘18	11	23	2.3	70
	‘19	11.6	25	5	108
Spring	‘18	18.5	29.6	6.4	133.2
	‘19	16	32.4	6	125

Unlike the male gametogenic line, the female germ line develops at the terminal ends of the follicles or acini.

The few oogonia present are characterized by being rounded, ∼10–15 µm in length, located in contact with the follicle walls, or attached to the latter by a pedicel, (Figs. 6A, 6B: oog). Those oogonia give rise to oocytes with condensed nuclei; which begin to accumulate yolk granules in the cytoplasm and, as they develop, increase in size to over 130 µm. The oogonia are always in direct contact with the walls of the follicle; and, only when they become mature oocytes they located in the lumen of the follicle or are found descending through the ducts.

Previtellogenic oocytes are rounded with a diameter between ∼20 and 60 µm, a basophilic cytoplasm without visible granules, a large nucleus, and a nucleolus (Fig. 4B: po). As the previtellogenic stages develop into vitellogenic oocytes, they measure between ∼60 and 100 µm and accumulate yolk progressively. Thus the cytoplasm changes to become eosinophilic (Fig. 4D: vo). Mature oocytes measuring ∼100–140 µm in diameter and possessing yolk granules, are observed in follicles at the pre-evacuation stage with more than six oocytes present in the gonad and descending towards the ducts (Figs. 5A; 6A–6D: mo). During the evacuation, up to three oocytes were descending through the ducts. During resorption (Figs. 7A, 7B) the follicles contained inner spaces or were collapsed and contained amaebocytes or phagocytic cells in the lumen (Fig. 7B: am).

### Gametogenic cycle

The gametogenic cycle can be divided into five stages:

Proliferation: This stage contains an abundant number of spermatogonia and some primary spermatocytes along with Sertoli cells. Follicles with oogonia and some previtellogenic oocytes (20–60 µm in length) are also growing.

Growth: This stage features the spermatogenic series with primary and secondary spermatocytes plus early and late spermatids. Few sperm cells are in contact with the epithelium of the follicle along with Sertoli cells. In the female germinal series, the oocytes (60–100 µm in length) are in vitellogenesis and with increasing amounts of yolk.

Pre-evacuation: In this stage, the lumen of the follicle contains abundant clusters of spermatozoa and late spermatids with their heads in contact with the epithelium of the follicle. Sertoli cells are present. Mature oocytes (100–140 µm in length) contain numerous yolk granules and are mostly located in the ducts of the follicles ready to be released.

Evacuation: The follicles contain few sperm clusters and Sertoli cells along with few oocytes filled with yolk. Some ovogonia begin to appear.

Resorption: The follicles become contracted or with a wide distance between them. In other instances, within the spaces in the lumen amaebocytes and developing spermatogonia are present along with some clusters of spermatozoa and unreleased oocytes.

**Figure 8:**
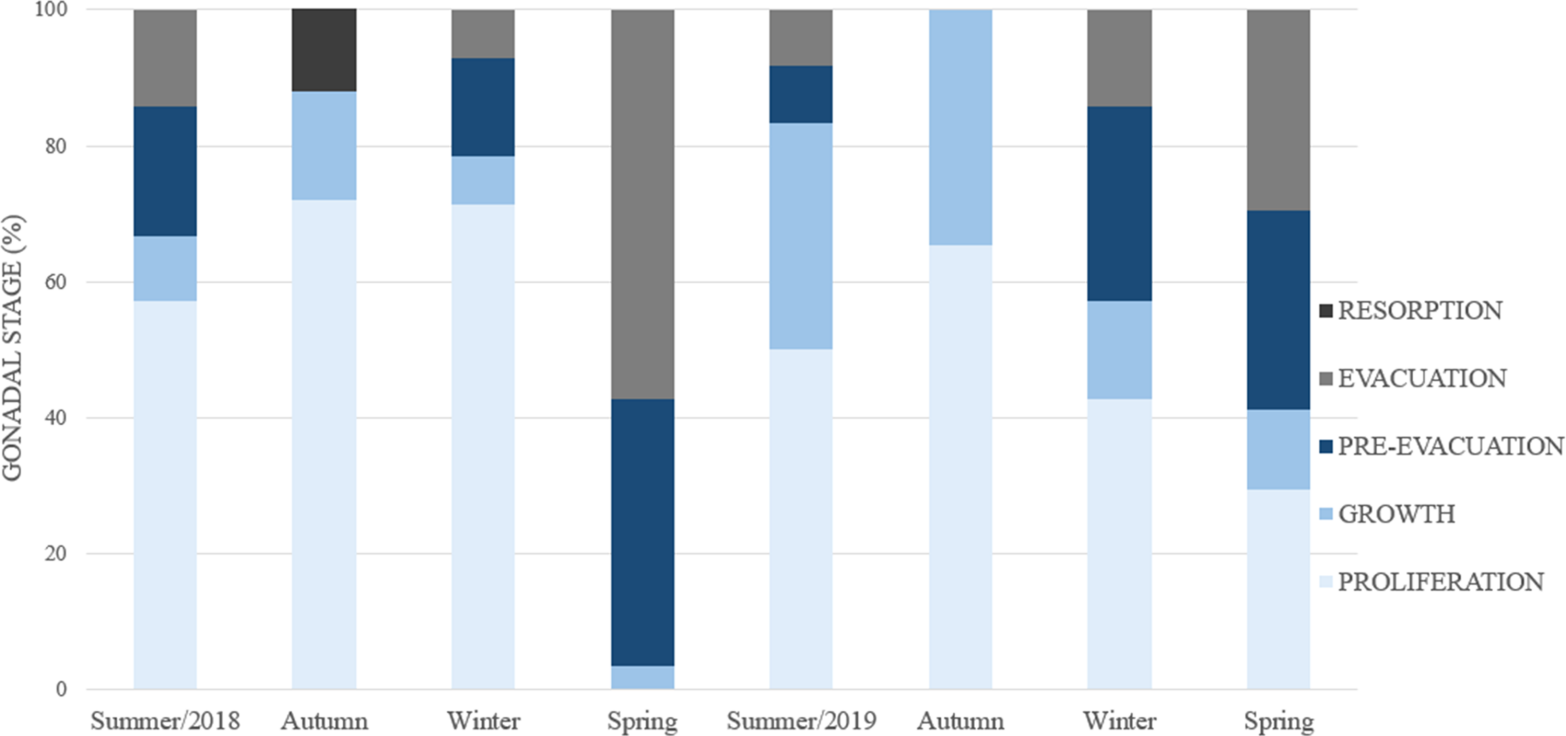
Percent frequency of the gonadal stages in *Bulimulus bonariensis* throughout the four seasons during 2018–2019. Image credit: Díaz Ana Carolina.

The seasonality of gonadal development (Fig. 8) reveals a continuous proliferation throughout the year, beging accentuated in summer, remains elevated in autumn and winter and decreases towards spring. No specimens were found in proliferation during the spring of 2018. The pre-evacuation and evacuation of gametes occurred mainly in the spring: these stages did not occur in autumn. Finally, resorption was only observed during the autumn of 2018.

### Periods of the gonadal cycle

The gonadal activity exhibited variations during the year that were separated into three periods:

Spawn: This stage began in winter when one part of the population was in gametogenesis and the other in spawning.

**Figure 9:**
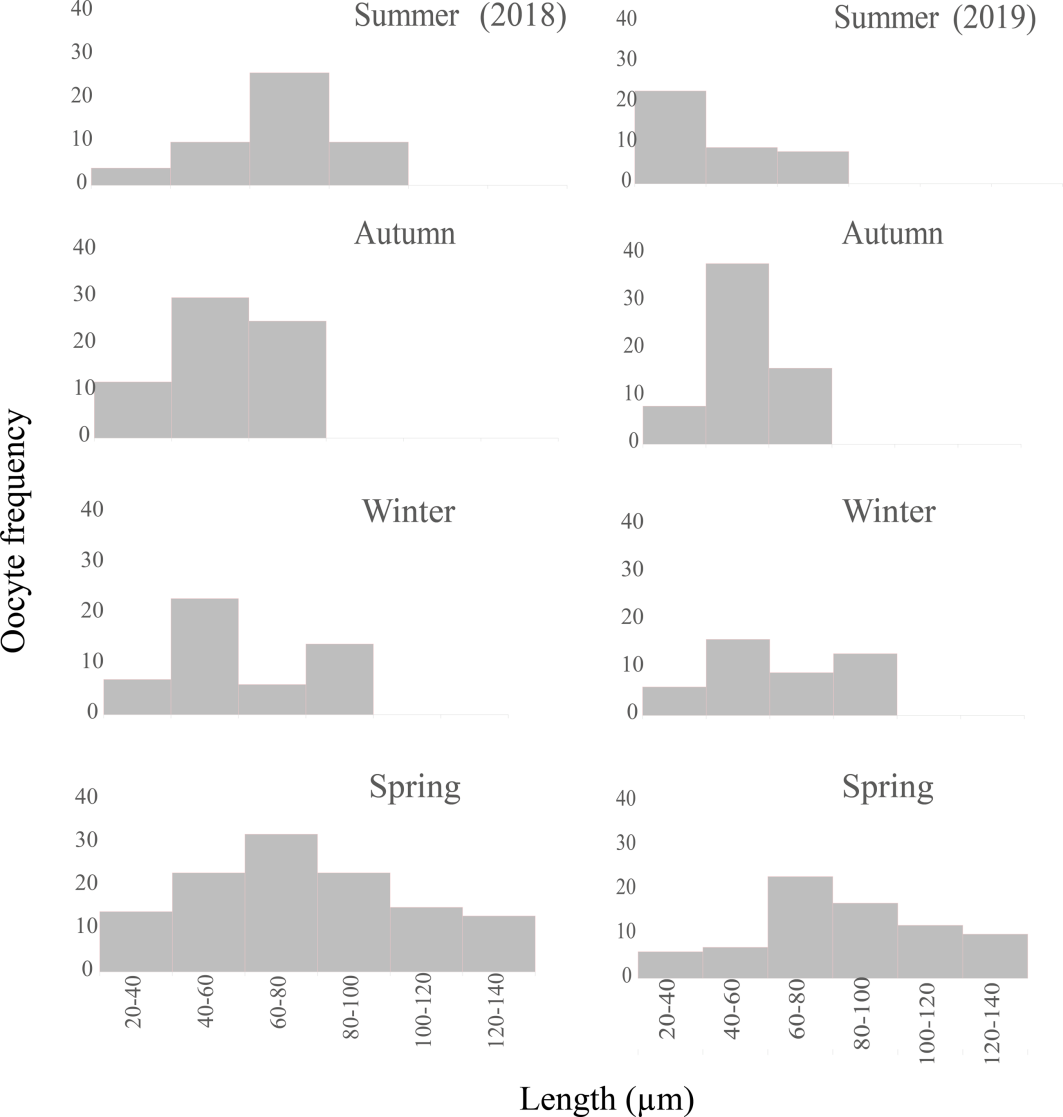
Seasonal oocyte size frequency distribution of *Bulimulus bonariensis* from March 2018 through December 2019. The figure illustrates the seasonal percent distribution of the oocyte size frequencies on the *ordinates* for 2018 (left column) and 2019 (right column) from the two summers above down through the two following springs below, with the key to the size ranges in µm represented by each bar indicated on the *abscissas* of the two spring distributions. Image credit: Díaz Ana Carolina.

**Figure 10:**
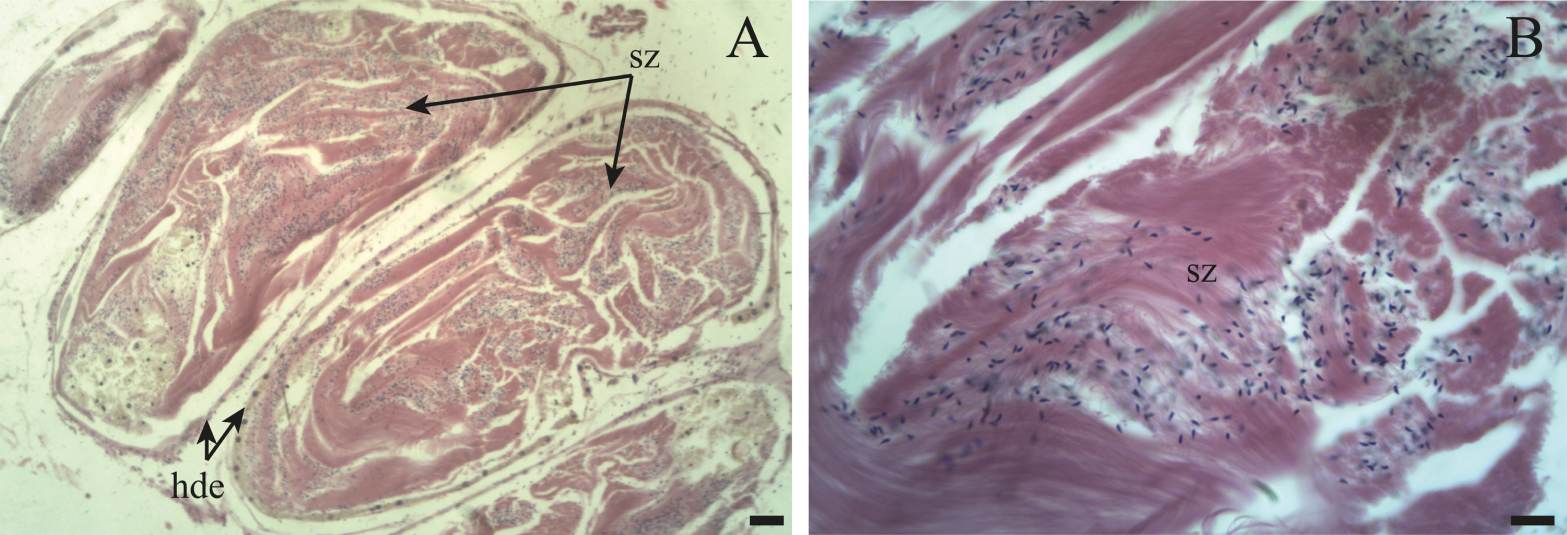
The hermaphroditic duct of *Bulimulus bonariensis*. (A) General view of the longitudinal section, featuring the hermaphroditic duct epithelium (hde) and spermatozoa (sz). Scale bar = 50 µm. (B) Detail of the spermatozoa (sz). Scale bar = 20 µm. Photo credit: Díaz Ana Carolina.

• Female germ line: during the spring, the germ line evidenced a proliferation of the oogonia, whereas during the rest of the year this cell type was not detected. In addition, the gonads contained oocytes of all sizes ranging from 20 to 140 µm (Fig. 9), few previtellogenic oocytes, increasing numbers of vitellogenic oocytes of 80–100 µm in length, and oocytes reaching the mature stage with lengths ranging from 100–140 µm were observed. Up to eight mature oocytes were found only in this season ready to be evacuated with abundant yolk granules (Figs. 6B, 6D). Thereafter, the size of the oocytes became smaller, indicating that the reproductive period occurred in this season.
• Male germ line: pre-evacuation was observed with several groups of mature spermatozoa in addition to spermatocytes and spermatids (Table 2). The ovotestis contained numerous clusters of sperm ready to be evacuated, while the hermaphroditic duct was likewise found to be replete with mature sperm (Fig. 10).

Post-spawn: this stage occurs in summer, with some individuals still in evacuation.

**Table 2:** Germ cell classification in *Bulimulus bonariensis*. Classification of germ cells: 0, absence of cell type; 0.5, with 1-2 cell clusters or 1-2 oocytes; 1, several cell clusters or 3-6 oocytes; 2, more than 10 cell clusters or more than six oocytes. Cell type: primary spermatocytes (sc1), secondary spermatocytes (sc2), early spermatids (st1), late spermatids (st2), mature spermatozoa (sz), previtellogenic oocytes (po), vitellogenic oocytes (vo), mature oocytes (mo).

Cell type	Year	Summer	Autumn	Winter	Spring
sc1	‘18	2	1	0.5	2
	‘19	1	2	0.5	0.5
sc2	‘18	2	2	2	2
	‘19	2	2	2	0.5
st1	‘18	2	1	1	2
	‘19	2	1	0.5	2
st2	‘18	2	1	1	1
	‘19	2	2	1	2
sz	‘18	2	1	1	1
	‘19	2	2	2	1
po	‘18	1	2	2	0.5
	‘19	2	2	1	0.5
vo	‘18	2	1	2	1
	‘19	1	1	2	1
mo	‘18	0	0	0	2
	‘19	0	0	0	2

• Female germ line: most of the oocytes measured were in the size range of 20–80 µm (Fig. 9), thus indicating the presence of a new generation of oocytes without a gonadal rest.
• Male germ line: in the spermatogenesis, the highest abundance of the five cell types was found.

Mature:

• Female germ line: the oogenic activity during the fall was observed with previtellogenic oocytes and abundant growing vitellogenic oocytes. The most frequent size range in the growing previtellogenic oocytes was 40–60 µm and in vitellogenic oocytes 60–80 µm, with the cells gradually incorporating yolk (Fig. 9).
  Resorption occurred only in the autumn along with the lysis of the oocytes and sperm that were not discharged, with gaps now being present between the follicles (Fig. 7A). In other specimens, follicles were observed with large lumens and amaebocytes (Fig. 7B). In winter, the most abundant previtellogenic oocytes were those ranging between 40 and 60 µm in length (Fig. 9).
• Male germ line: the highest abundance of primary and secondary spermatocytes was found in autumn. Although the male germ line proved have continuous spermatogenic activity throughout the year, with numerous clusters of spermatogonia to initiate the next gametic generation; all the stages were present throughout the two years of the study. In winter, as mentioned above, that part of the population evidenced a great proliferation with abundant secondary spermatocytes plus early and late spermatids.

### Morphologic variation in relation to the gametic cycle

**Figure 11:**
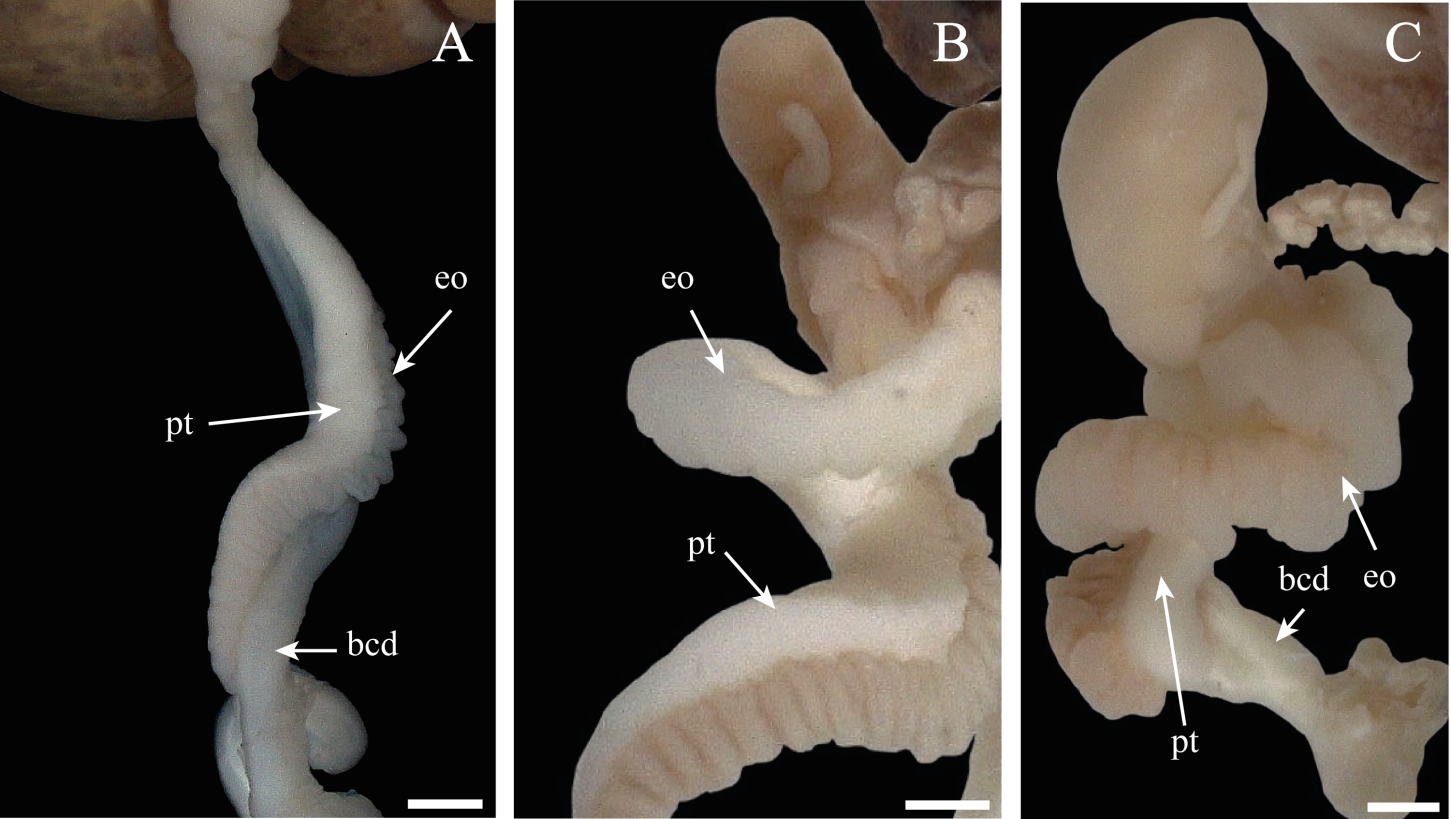
Morphologic variation of the spermioviduct of *Bulimulus bonariensis*. (A) Undeveloped spermioviduct (eo) of adult specimens. (B) The developing spermioviduct. (C) The developed spermioviduct is present plus the duct of the bursa copulatrix (bcd) and the prostate (pt). Scale bar = 1 mm. Photo credit: Díaz Ana Carolina.

The only structure that underwent periodic anatomical variations was the spermioviduct, which passed through three phases corresponding to the successive stages of gametic development, being thin with an undeveloped appearance and not folded; during development, manifesting an intermediate diameter with slight folding; and, finally, containing a spermioviduct with an appreciable degree of development and folded back on itself (Fig. 11). When we compared these characteristics and the stage in which the gonad was found, we were able to recognize the following:

**Figure 12:**
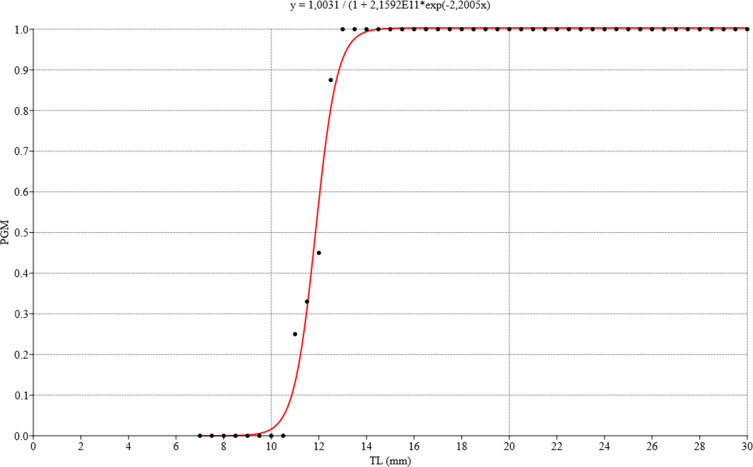
Logistic regression model for estimating gonadal maturity of *Bulimulus bonariensis*. Logistic regression model applied to the proportion of gametic maturity (PGM) as a function of the total shell length (TL), expressed in millimeters (0 = undifferentiated and/or immature, 1 = mature). Image credit: Díaz Ana Carolina.

• Some specimens evidenced undeveloped or thin spermioviducts corresponding to proliferating or growing stages of gonad (Fig. 11A).

• Other specimens contained spermioviducts in the process of development with an intermediate diameter correspond to the Maturity stage of gonadal development (Fig. 11B).

• Still other specimens exhibited well developed spermioviducts corresponding to the Spawning or evacuation, Post-Spawn and reabsorption stages of gonadal development (Fig. 11C).

Therefore, the stage of development of the spermioviduct was found to be clearly an informative element in determining, *a priori*, the stage of development of the ovotestis.

### Size at first maturity

The ovotestis was observed at different stages of development, namely, undifferentiated with no germ-cell differentiation (Fig. 3A), followed by immaturity or gametogenic activity and later maturity. We also observed that the first previtellogenic oocytes appeared from the time associated with a total shell length of 8.42 mm, while the first spermatogonia were observed at a shell length of 9.92 mm. Figure 12 illustrates the distribution of the proportion of gametic maturity that corresponds to the total shell size (TL). The data indicated a good fit to the model with the following pseudo-R squared: Cox and Snell = 0.732, Nagelkerke = 1.

The size at which 50% of the population was considered mature was approximately 12 mm TL.

**Figure 13:**
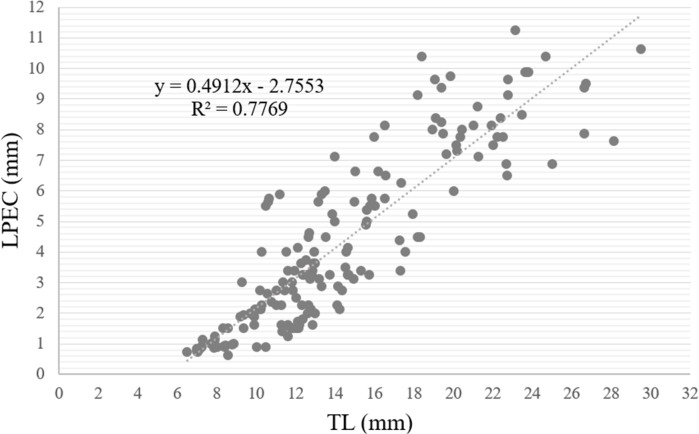
Length of penile complex (LPEC) of *Bulimulus bonariensis* as a function of total shell length (TL). In the figure, the length of the penile complex (LPEC) in µm is plotted as a function of the total shell length in µm. The line through the points was generated by the equation in the upper left of the figure for the best fit of the data.

When the length of the penile complex (LPEC) was plotted as a function of the TL of the specimens (Fig. 13), the correlation between both variables was positive and statistically significant (*r* = 0.88, *p* < 0.01). In addition, we observed that in individuals larger than 13 mm, when they had already reached gonadal maturity, the dispersion was greater, where 81.7% of the instances corresponded to individuals with an LPEC of four millimeters or more.

## Discussion

The general structure of the gonad and the parallel arrangement of the acini or follicles of *B. bonariensis* was consistent with what has been observed in other gastropods, for example: *Cornu aspersum* (Müller, 1774), *N. major* and *H. obvoluta* (Griffond & Bolzoni-Sungur, 1986; Cuezzo, 1990; Maltz, 2003).

De Jong-Brink, Boer & Joose (1983) in pulmonates, Griffond & Bride (1987) in *C. aspersum* and Luchtel (1972) in *Arion circumscriptus* Johnston, 1828 indicated that the gonadal follicles were divided into a central compartment, corresponding to the lumen, where the male cells develop, and a peripherally positioned female region. Cuezzo (1993) observed a tendency to this regionalization in *S. tupacii*. In *B. bonariensis*, however, the development of both germ lines, the spermatogonia and the oogonia, have a location in close association with, or at least joined by a pedicel to, the follicular epithelium; as was reported by Ngowsiri et al. (1989) for *L. fulica*. When the oogonia proliferated and grew, they remained in contact with this epithelium and only packets of spermatozoa and oocytes were observed occupying the medullary region of the follicle upon becoming mature. In a different approach, Griffond & Bride (1987) sectored each follicle into three rings: a distal end where the sex cells developed, an intermediate germinal ring, and a follicle neck. In *B. bonariensis* the arrangement of the female germ line was observed entirely at the distal end of the follicle, with the male germ line being likewise at the distal end but also along most of the follicle.

The observations of the histological sections evidenced that, in the smallest individuals, the female gametes differentiated earlier than the male gametes, with the first to appear being the previtellogenic oocytes. For this reason, we could conclude that, as Luchtel (1972) had observed in *A. circumscriptus*, the individuals were initially protogynous hermaphrodites, an uncommon reproductive strategy in mollusks (Zajac & Kramarz, 2017) and one entirely unlike that of another representative of the Bulimulidae family, *S. tupacii* (Cuezzo, 1993), where the male gametes mature earlier than the female. Then, upon reaching gonadal maturity, both spermatozoa and mature oocytes are present, thus becoming simultaneous hermaphrodites as observed in many other terrestrial gastropods such as *A. circumscriptus*, *A. ater*, *N. major*, *S. tupacii*, *M. abbreviatus*, *Vestia gulo* (Bielz, 1859), *V. turgida* (Rossmässler, 1836), and *Cerion mumia chrysalis*, among others (Luchtel, 1972; Parivar, 1978; Cuezzo, 1990; Cuezzo, 1993; Horn, Achaval & Zancan, 2005; Maltz & Sulikowska-Drozd, 2010; Suarez, 2015). In addition, we were able to determine that the reproductive strategy of *B. bonariensis* is of the iteroparous type, the most common in terrestrial gastropods (Zajac & Kramarz, 2017).

As to the gametic development, the oogonia could be observed only during the Spawning period in the evacuation stage of individuals, indicating that the proliferation of this germline occurs only once a year. Throughout the annual cycle, only oocyte growth and development were observed. Abundant clusters of spermatogonia were found throughout the annual cycle, indicating continuous proliferation. Spring was the period when the mature spermatozoa and oocytes were found in the ovotestis of *B. bonariensis* with the recruitment of a new generation of individuals also taking place at that time. The simultaneous presence of mature oocytes, a lower relative abundance of mature spermatozoa in the gonad, and a hermaphroditic duct replete with the latter indicated that the main copulation period would occur during the spring. In *H. pomatia* (Lind, 1973), *H. lutescens* Rossmässler, 1837 (Koralewska-Batura, 1999), *M. abbreviatus* (Horn, Achaval & Zancan, 2005), and *O. spectabilis* (Ferreira et al., 2012); the main breeding period was also observed at this season, unlike the gametogenic cycle of *S. tupacii* (Cuezzo, 1993) and *Leptaxis caldeirarum* (Morelet & Drouet, 1857) (Rodrigues & Medeiros, 2005); which species breed during the summer.

At the end of the Spawning period, during the summer and especially in the fall, high levels of proliferation and growth were observed in individuals in the evacuation stages. The observations indicated that this species immediately started a new gametic generation for reproduction in the following spring; as had also been observed in two species of *Vestia* by Maltz & Sulikowska-Drozd (2010), in *M. abbreviatus* by Horn, Achaval & Zancan (2005), and in *V. pusilla* by Mazurkiewicz & Pokryszko (2005). Therefore, these species have continuous gonadal activity, without an annual rest period. In addition, the continuity of the gamete production cycles was evidenced by the presence of both germ lines in all four seasons involving only variations throughout the year and with the higher relative abundance of all cell types of the male germ line during the summer, the Post-spawning period. The oocyte size histograms (Fig. 9) evidenced how the number of oocytes and their modal lengths varied according to the stage of development. Moreover, the identification of mature sperm throughout the year would indicate that the Spawning period was determined by oocyte maturation, an observation also reported by Maltz (2003) in *H. obvoluta*.

According to Maltz & Sulikowska-Drozd (2008), two maturation strategies exist: those species in which sexual maturity is reached at the same time as the maximum size, as occurs in Helicidae; and those in which, after reaching sexual maturity, the snail continues to grow, as occurs in Succinidae and Vitrinidae. *Bulimulus bonariensis* is included in the second of these strategies, since after reaching maturity (at a 12 mm TL and a four millimeters LPEC), those pulmonates continue to grow.

In contrast, in certain species of gastropods, for example *Melongena corona* (Gmelin, 1791) (Zetina Zárate et al., 2000), the macroscopic characteristics of the gonad such as the size, coloration, and appearance can be used to differentiate the different age classes. In the male gonad of *Zidona dufresnei* (Donovan, 1823), changes in coloration were reported (Roche, 2013), and in *S. tupacii* (Cuezzo, 1993) variations in both size and coloration have been observed throughout the gametic cycle. *B. bonariensis* manifested no such characteristics, but the degree of development of the spermioviduct was found to be a useful structure for predicting the stage of gonad maturity. In addition to this anatomical feature, we were able to establish a minimum shell length and size of the penile complex at which individuals of this species reach gonadal maturity. The aforementioned data constitute essential information for determining, *a priori*, the degree of development in animals collected in rural areas where the presence of this species has generated different economic problems (Frana & Massoni, 2007; Frana & Massoni, 2011).

The reproductive activities of gastropods from different families exhibit both similarities and differences between the groups that are not related through phylogenetic proximity, but rather depend on the characteristics of the environment. The most influential environmental parameters for terrestrial gastropods are the temperature, the humidity, the rainfall frequency, the lengths of dry and wet seasons, the duration of light, the photoperiod, the type of vegetation, and the availability of water and food. These exogenous components act by modelling endogenous networks related to metabolic processes such as the maturation times of the reproductive system, the hibernation and/or estivation periods, the length and seasonality of the reproductive season, the speed of gonadal development, the proliferation of germ cells -especially at the intervals of transition from secondary spermatocytes to early spermatids- and the attainment of maturity (Luchtel, 1972; Bailey, 1981; De Jong-Brink, Boer & Joose, 1983; Ngowsiri et al., 1989; Fontanillas, García-Cuenca & Pérez Fuentes, 1992; Cuezzo, 1993; Koralewska-Batura, 1999; Healy, 2001; Barrada Beiras, 2003; Terwissen et al., 2007; Maltz & Sulikowska-Drozd, 2008; Maltz & Sulikowska-Drozd, 2010).

Possibly, reproduction in spring was favored by the climatic characteristics of the study site. In this season, temperatures oscillate around 17 °C and rainfall varies little from one year to the next, with an average of 129 mm. This temperature is in the optimal range for reproduction, according to the temperature ranges given by Li et al. (2021) for other land snail species. In addition, the humidity of this period of year encourages individuals to dig themselves up, reproduce, the eggs do not dry out, and therefore hatch. Although in summer some snails were found in gamete evacuation, and therefore in spawning. Severe seasonal heat waves are very likely to cause egg dehydration and embryonic death. Consequently, temperature is the main factor controlling egg hatching, followed by drought or low of precipitation (Li et al., 2021). In this context, we must consider the wide distribution of the species and its presence in various climates, such as humid subtropical (Corrientes, Misiones, Santa Fé), serrano subtropical (Tucumán), subtropical with dry season (Santiago del Estero), humid subtropical with dry season (Chaco, Formosa), temperate humid (Buenos Aires, Entre Rios, Córdoba) and dry tropical (Salta, Jujuy). These climates have different temperatures, thermal amplitudes and different rainfall regimes. Thus, the information provided in this study can be used to interpret and establish comparisons with populations living in other areas of Argentina with a different climate.

The present study carried out on *B. bonariensis* enabled a determination of the time of the year in which the Spawning period took place in order to establish the minimum total length of the penile complex at which the individuals reached gonadal maturity. These findings also contribute to a more comprehensive understanding of the basic aspects of this snail’s biology.

##  Supplemental Information

10.7717/peerj.15221/supp-1 Supplemental Information 1 Measurements in information associated with *Bulimulus bonariensis* specimens collected in the period 2018–2019

10.7717/peerj.15221/supp-2 Supplemental Information 2 Seasonal oocyte size frequency distribution of *Bulimulus bonariensis* 2018–2019

## Additional Information and Declarations

### Field Study Permissions

The following information was supplied relating to field study approvals (i.e., approving body and any reference numbers): Sampling was carried out with authorization through an agreement between the UNLP and the Municipality of Florencio Varela.

### Data Availability

The following information was supplied regarding data availability: The raw measurements are available in the Supplementary Files.
